# Convergent mechanisms, divergent strategies: a comparison of nectar intake between a generalist and a specialist bat species

**DOI:** 10.1242/jeb.251404

**Published:** 2026-03-11

**Authors:** Laura L. Quinche, Felipe Garzón-Agudelo, Sharlene E. Santana, Hugo F. López-Arévalo, Alejandro Rico-Guevara

**Affiliations:** ^1^ Department of Biology, University of Washington, Seattle, WA 98195, USA; ^2^ Centro de investigación Colibrí Gorriazul, Fusagasugá, Cundinamarca, 252217 Colombia; ^3^ Burke Museum of Natural History and Culture, Seattle, WA 98195, USA; ^4^ Grupo en Conservación y Manejo de Vida Silvestre, Instituto de Ciencias Naturales, Universidad Nacional de Colombia, Bogotá, 111321 Colombia

**Keywords:** Phyllostomidae, Functional morphology, Feeding kinematics, Feeding performance, Lapping, Tongue papillae

## Abstract

Nectar-feeding bats exhibit a range of specialized adaptations that allow them to extract nectar from flowers efficiently. These adaptations include tongue morphological traits and feeding strategies that reflect varying degrees of specialization to nectarivory. While some aspects of the drinking mechanics of highly specialized nectar bats have been studied, little is known about the feeding behaviors of non-specialized species such as *Phyllostomus discolor*. This study compares the nectar extraction behaviors of *P. discolor* and the specialized *Anoura geoffroyi*, examining morphological and biomechanical adaptations that affect feeding efficiency and foraging strategies. We used electron microscopy to study the lingual surface, and high-speed videography to analyze tongue kinematics and feeding efficiency. Both bat species possess hair-like papillae that form a brush-like tongue surface, and both extract nectar using a lapping mechanism; however, they exhibited notable behavioral and biomechanical differences resulting in variation in feeding efficiency. *Phyllostomus discolor* has a shorter, less flexible tongue than *A. geoffroyi*, but exhibits similar licking frequencies. Unlike *A. geoffroyi*, which performs brief hover-feeding bouts, *P. discolor* perches on the inflorescences, drinks for longer, and extracts more nectar per visit. However, *P. discolor* exhibited lower feeding efficiency, likely due to its reduced tongue protrusion distance and shorter, less abundant papillae. These findings reveal convergence in general feeding mechanism, i.e. brush-tongue lapping, but highlight divergence in morphological and behavioral traits that affect feeding kinematics and efficiency. Our study illuminates how foraging strategy and tongue morphology affect drinking efficiency, pointing to evolutionary pathways that promote niche differentiation within nectar-feeding bat communities.

## Teaser

**Highlighted Article:** Nectar-feeding bats converge on brush-tongue lapping but diverge in tongue morphology and foraging strategy, driving differences in feeding kinematics and efficiency.

## INTRODUCTION

Nectar is one of the most ubiquitous food resources and it mediates mutualistic relationships between plants and animals (Nicolson et al., 2007). Flower visitors consume this highly nutritious reward produced by plants and participate in plant pollination by transporting and transferring pollen (Nicolson et al., 2007; Parachnowitsch et al., 2019). There is a wide diversity of nectar consumers, from jumping spiders to bats (Fleming and Muchhala, 2008; Jackson et al., 2001), with varying degrees of nectarivory that span an ecological continuum from opportunistic to highly specialized flower visitors.

Within the diverse order of bats (Chiroptera), the nectarivorous diet has evolved independently in Paleotropical fruit bats (Pteropodidae), Neotropical leaf-nosed bats (Phyllostomidae) (Fleming et al., 2009) and in two species of the families Vespertilionidae (*Antrozous pallidus*; Frick et al., 2014) and Mystacinidae (*Mystacina tuberculata*; Arkins et al., 1999). Within Phyllostomidae, nectarivory evolved independently as the main feeding habit in three lineages: the Glossophaginae and Lonchophyllinae subfamilies and the *Phyllostomus* genus (Rojas et al., 2011). Although nectar and pollen constitute major dietary components in species from all three lineages, none are strictly nectarivorous, and dietary breadth as well as nectar-feeding adaptations vary within and among taxa. In fact, the most specialized nectar-feeding vertebrates (hummingbirds; Hewes et al., 2023) are not strictly nectarivorous; they fulfill their protein requirements by hunting a variety of arthropods (Rico-Guevara, 2008; Stiles, 1995). Glossophagine and lonchophylline bats are characterized by pronounced morphological, behavioral and physiological specializations associated with nectar feeding, including hovering flight, elongated rostra and highly extensible tongues with specialized papillae (Fleming et al., 2009; Harper et al., 2013). In contrast, species of the genus *Phyllostomus* are generally considered to be more omnivorous (Gardner, 2008) and lack many of these phenotypic specializations for nectar feeding (with some exceptions discussed in detail below); nevertheless, they can rely heavily on floral resources. For example, *Phyllostomus discolor* exhibits a high level of nectarivory in its diet (82.1% pollen and nectar: Kalko et al., 1996; 70.4% pollen: Giannini and Kalko, 2004), based on analyses of fecal samples and food remains representing total diet composition.

A bat extracts nectar from a flower by bringing its snout to the corolla entrance and extending its tongue into the nectar chamber; thereby, the tongue becomes the primary food acquisition organ. Researchers have sought to elucidate the importance of the tongue during feeding in specialized nectarivorous bats by characterizing tongue morphology (Greenbaum and Phillips, 1974; Howell and Hodgkin, 1976; Griffiths, 1978; Birt et al., 1997; Nicolay and Winter, 2006) and nectar-feeding behavior (Bechler et al., 2024; Gonzalez-Terrazas et al., 2012; Harper et al., 2013; Tschapka et al., 2015) and relating these traits to foraging efficiency (Gonzalez-Terrazas et al., 2012; Nicolay and Winter, 2006; Bechler et al., 2024; Gamba et al., 2025). In specialized nectarivorous bats, two different morphological and biomechanical strategies have been observed for extracting nectar from flowers. *Glossophaga soricina* has lingual hair-like (filiform) papillae and employs a lapping mechanism in which the papillae are erected by blood flow (brush-tongue lapping technique) (Harper et al., 2013; Tschapka et al., 2015). In contrast, *Lonchophylla robusta* has lateral grooves in the tongue, and its extraction mechanism consists of pumping nectar into the mouth through the lingual grooves (pumping-tongue drinking technique) (Tschapka et al., 2015). Little is known about the morphology associated with nectarivory in non-specialized species (e.g. *Phyllostomus* spp.; Nicolay, 2001; Nicolay and Winter, 2006; Quinche et al., 2023) and the nectar extraction mechanism in these bats has not been described. Examining the morphology and nectar uptake behavior of non-specialized species is essential to determine whether additional modes of nectar extraction have evolved in bats, and could shed light on the initial steps to specialized nectarivory within the lineage.

Morphological and behavioral traits related to nectar consumption have been recognized in *P. discolor*, such as a long and highly mobile tongue with hair-like papillae on its surface (Quinche et al., 2023). Additionally, it has been shown that *P. discolor* can achieve nectar extraction rates comparable to those of *G. soricina* (Nicolay, 2001; Nicolay and Winter, 2006). However, a complete description and quantitative study of *P. discolor* nectar uptake behavior, as well as a direct, experimental comparison with specialized species, is still lacking. Filling this knowledge gap is important for better understanding the relationship between feeding behavior and efficiency in species with varying degrees of morphological and ecological specialization. In this study, we conducted experiments to compare the non-specialized *P. discolor* with a nectar specialist glossophagine, *Anoura geoffroyi*. Previous research has described morphological adaptations to nectarivory in *A. geoffroyi*, such as a highly protrusible tongue with hair-like papillae (Bhatnagar and Smith, 2007; Muchhala et al., 2024), but no studies have examined the extraction mechanism and efficiency for either of these species.

The nectar extraction mechanism employed by the genus *Phyllostomus* is not yet known, but we hypothesize that it resembles the brush-tongue lapping technique of glossophagine species for two main reasons. First, from an evolutionary perspective, lapping is the typical mode of liquid ingestion in adult mammals, especially in species with incomplete cheeks that cannot form a tight oral seal and therefore cannot generate negative pressure for suction (Williams, 2019). More elaborate mechanisms, such as those seen in Lonchophyllinae, would have evolved later in response to particular selective pressures during plant–pollinator interactions, e.g. the evolution of long corollas and tongues that can extract the nectar deep inside without spending extra time reciprocating. Second, from a morphofunctional perspective, we expect *P. discolor* to use its tongue similarly to glossophagines, as the two taxa share hair-like papillae and lack the groove-like structures on the sides of the tongue found in lonchophyllines.

Given the considerable differences in size (*P. discolor* 39.7–44.6 g and *A. geoffroyi* 8.5–13.0 g; Kwiecinski, 2006; Oprea et al., 2009), foraging strategy, diet specialization and evolutionary history, we predicted that even though the mechanism could be similar (brush-tongue lapping technique), there would be clear dissimilarities in nectar feeding behavior between these species. Specifically, we expected *P. discolor* to exhibit longer visits as it lands to feed (Giannini and Brenes, 2001; Pedrozo et al., 2018), as opposed to *A. geoffroyi*, which would hover while feeding and therefore would also be limited by the energy invested in this expensive flight style (Voigt, 2004; Voigt and Winter, 1999). Lastly, we predicted that the licking frequency, amount of nectar extracted and drinking efficiency would be higher in *A. geoffroy*i because of its more specialized feeding habits and tongue morphology.

## MATERIALS AND METHODS

### Field survey, captivity and specimen collection

Fieldwork was conducted in two different locations and periods: (1) in Hacienda La Cabaña (Sede Cabaña), Cumaral, Meta, Colombia, between May and June 2019, and (2) in Colibrí Gorriazul Research Center, Fusagasugá, Cundinamarca, Colombia, in October 2019, February 2021 and January 2023. Bats were captured with mist nets, and all bats were released, except for adult *Phyllostomus discolor* Wagner 1843 (*n*=10) and *Anoura geoffroyi* Gray 1838 (*n*=11) individuals, which were held in captivity in a flight cage (2.5×2.5×2.5 m) for experiments. Female bats were confirmed to be neither pregnant nor nursing. Between both species, we kept a maximum of four individuals in the cage at the same time. We did not see any aggressive interactions among individuals while sharing the cage during the experiments. In the cage, the animals could fly freely and hang from the walls and ceiling. We placed one or two feeders designed like a banana inflorescence (a common local food source known to be visited by both species), with a hemispherical cone shape approximately 30 cm long, which allowed the bats to hang while drinking nectar as needed (Fig. S1). We attached a transparent flat-sided container (nectar container: 11 mm×11 mm×85 mm) to one of the feeders.

After capture, the bats were habituated to the experimental setup inside the cage (nectar container and cameras/lights) by recording videos of them drinking nectar while the bat was hand-held. The position of the feeder was kept constant, and it was shown to the bats by releasing them above the feeder. The bats were trained for 1 night, after which all of them learned to visit the nectar container by flying to it on their own and returning to their preferred hanging spot.

During captivity, bats were provided with artificial nectar, fruit and water *ad libitum* while in the cage, although consumption of fruit and water was not quantified. Every 1 or 2 nights, bats were taken from the cage to individually feed them a mixture of artificial nectar with crushed mealworms before being released back into the cage. Individuals spent 2–4 nights in captivity, after which they were released at the capture site. We collected two adult, non-pregnant, non-lactating female individuals as voucher specimens: one of *P. discolor* during fieldwork in Cumaral, Meta, Colombia, and one of *A. geoffroyi* during fieldwork in Fusagasugá, Cundinamarca, Colombia. The individuals were held in captivity and collected under the institutional collection permits of the Universidad Nacional de Colombia (number: 0255, March 2014). We followed the IACUC guidelines recommended by the American Society of Mammalogists for all procedures (Sikes and the Animal Care and Use Committee of the American Society of Mammalogists, 2016), which are equivalent to the ones followed by the Universidad Nacional de Colombia's permits.

### Tongue morphology

To describe and compare the lingual surface of the two species, we dissected the tongues from the two specimens collected, and prepared them for scanning electron microscopy (SEM). First, we fixed the tongues in 4% formaldehyde, dehydrated the samples and covered them with a gold coating. We obtained the SEM images from the Laboratorio de Microscopía Electrónica de Barrido at the Universidad Nacional de Colombia with an FEI Quanta 200 scanning electron microscope (FEI Company, Eindhoven, The Netherlands) and from the Laboratorio de Microscopía Electrónica de Barrido at the Pontificia Universidad Javeriana de Colombia with an EVO HD15 scanning electron microscope (Zeiss, Jena, Germany); these scanning electron microscopes were operated at an accelerating voltage of 30 and 10 kV with direct magnifications up to 1000×.

### Nectar extraction experiment design

Bats were recorded visiting the nectar container (85 mm in height, 11 mm internal width) filled with artificial nectar (sucrose solution at 17% w/w concentration) at different distances from the upper rim (i.e. nectar depth), which varied as the nectar was depleted. This concentration was chosen to maintain consistency with previous studies (Gonzalez-Terrazas et al., 2012; Tschapka et al., 2015). *Phyllostomus discolor* and *A. geoffroyi* individuals were filmed with high-speed video cameras (JVC GC-PX100BU, Fastec TS5, and Chronos 1.4, Krontech) set at 240–1000 frames s^−1^. We made videos with two main lighting configurations: LED light at the back or side of the tube and infrared (IR) lights from the outside of the feeder setup, aimed at the feeder. We focused the camera on the test container using tele-macro modes/lenses. We recorded two types of high-speed videos: (1) videos of hand-held individuals drinking nectar in different views (lateral, frontal and ventral; ‘hand-held’ videos) and (2) videos of free-flying individuals inside the cage approaching volitionally to drink nectar (‘free-flying’ videos).

### Video data collection

We used ImageJ-Fiji (v2.14.0; National Institutes of Health, Bethesda, MD, USA) to quantify variables from high-speed videos. To characterize tongue movements, we tracked the tongue tip movements of nine visits of four *P. discolor* individuals and 17 visits of five *A. geoffroyi* individuals feeding from the test container at nectar depths ranging from 8 to 16 mm, defined as the vertical distance from the container rim to the nectar surface. To facilitate species comparisons, we analyzed tracking data from the first lick of each visit in acclimated bats that had previously fed from the nectar container, focusing on cases where the tongue made contact with nectar. We selected the first lick to standardize comparisons across visits and to capture tongue–nectar interactions before potential modulation as a result of sensory feedback, nectar depletion or fluid movement within a feeding bout. As lick duration varied within species, we normalized it to a percentage scale, with 0% marking the start and 100% the end. We then calculated and plotted the mean first lick pattern for each species.

To characterize the general drinking behavior of both species (beyond tongue kinematics), we obtained videos of 40 visits of 10 *P. discolor* individuals and 88 visits of 11 *A. geoffroyi* individuals feeding at a wider range of nectar depths (from 0 to 32 mm). The larger sample size for *A. geoffroyi* relates to more frequent, but shorter, visits (see Results). We measured the licking time as the time from the first appearance of the tongue tip (the start of licking) until the moment the tongue was entirely retracted into the mouth (the end of licking), regardless of the total number of licks. In all of these visits, the tongue contacted the liquid at each lick. To obtain licking time values, the frame count was converted to seconds according to the corresponding frame rate. We measured the snout and tongue insertion into the test container (from the entrance to the tip of the snout/tongue in mm). The amount of nectar extracted was calculated by measuring the change in the nectar meniscus in the container before and after each visit, converting this change to extracted volume using the container dimensions, and then converting volume to mass using the density of the sucrose solution to allow comparison with previous studies. Nectar extraction per lick was calculated for each visit as the total volume of nectar extracted divided by the number of licks during that visit. We calculated nectar extraction efficiency, *E* (g s^−1^), by dividing the amount of nectar extracted by the total licking time. Then, for a more meaningful comparison between species of different sizes, which would reflect their different energy demands, we calculated the size-adjusted efficiency *E*_s_ (g s^−1^ g^−1^) as follows:
(1)

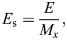
where *M_x_* is the body mass of species *x* in grams. As multiple individuals were present in the cage simultaneously and individual visits to the feeder could not be tracked, we used the average body mass of the individuals captured per species.

The mean maximum tongue protrusion length (mm) was measured from the snout tip to the tongue tip along the dorsal surface of the tongue, taken from lateral videos, and defined as three consecutive attempts to reach deeper nectar without success and with minimal change in tongue extension. Maximum tongue protrusion differs from tongue-tip insertion in that maximum tongue protrusion is a tongue-only metric that follows the curved surface of the tongue; in contrast, tongue-tip insertion is a tube-based metric measured as a straight distance along the tube and reflects how far the tongue tip is positioned inside the tube. Because this straight distance is referenced to the tube opening, it also reflects the degree to which the snout enters the tube, without separating snout and tongue contributions. Tongue immersion was measured as the maximum length of the tongue in contact with nectar during a lick. Specifically, we measured the distance along the dorsal surface of the tongue from the tongue tip to the most proximal point where the tongue was still in contact with the nectar surface. Finally, we calculated licking frequency (licks s^−1^) by dividing the number of licks over the time elapsed.

### Statistical analysis

We used generalized linear models (GLMs) to explore the effects of species and nectar depth as explanatory variables on various response variables that describe drinking behavior. The response variables were: the number of licks, the amount of nectar extracted, licking time, nectar extraction efficiency, standardized extraction efficiency, lick frequency and maximum tongue extension. We employed the Poisson distribution and log link function for the dependent variable ‘number of licks’ and the Gamma distribution and the inverse link function for the rest of the dependent variables. Statistical tests were performed using the ‘glm’ function from the stats R package (v4.2.3; http://www.R-project.org/).

## RESULTS

### Overall behavior during feeding

Once the bats were released into the cage, after being hand-held fed at the feeder with the nectar container, they flew in circles and explored the surrounding space before approaching the container again. All captive individuals learned the location of the nectar source and began visiting the feeders on their own on the first or second night. *Phyllostomus discolor* always landed on the feeders before drinking. First, they landed on different parts of the feeder to then crawl towards the nectar container, and after some visits, they landed directly in front of the container. They landed near the top of the feeder and hung upside down, sometimes keeping their wings open and other times folding them while drinking nectar. Conversely, *A. geoffroyi* always hovered to feed from the nectar container, making quick, consecutive visits.

Although *P. discolor* individuals usually drank nectar until they seemingly could not drink any more (because they could not reach deeper into the container, e.g. Movie 1), *A. geoffroyi* individuals spent almost the same time during each visit, reaching deeper. Both species rarely closed their eyes while feeding, and no territorial behavior was observed around the feeders. When more than one individual of the same species was in the cage, the bats preferentially perched together when they were not feeding and left these roosting sites to forage together, often taking turns to access the nectar. We observed this joint foraging behavior more frequently in *A. geoffroy*i than in *P. discolor*.

### Tongue kinematics and morphology

**Fig. 1.**
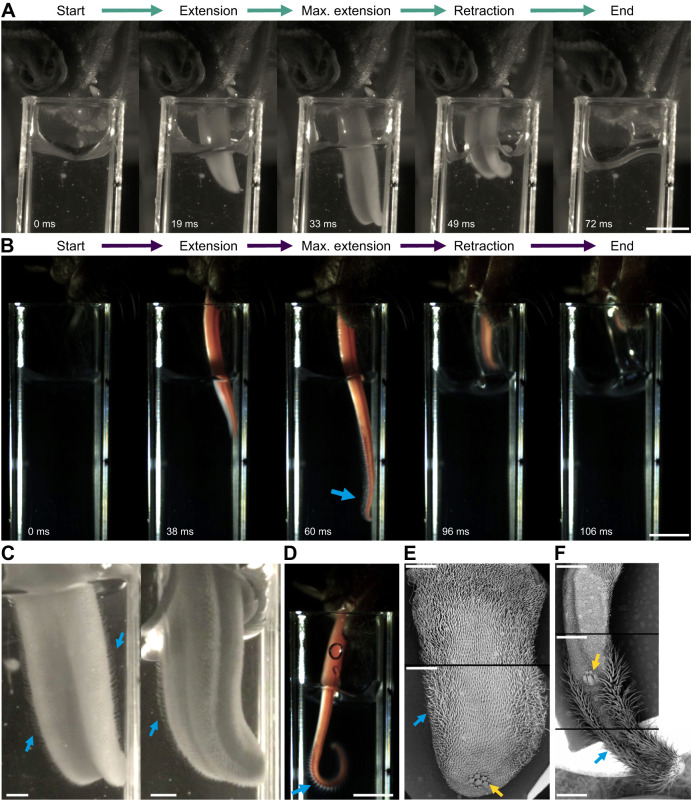
**Lapping mechanisms and hair-like papillae of *Phyllostomus discolor* and *Anoura geoffroyi*.** (A,B) Frames from a high-speed free-flying video showing the nectar extraction sequence of a single lick in *P. discolor* (A) and *A. geoffroyi* (B). The blue arrow points at hair-like papillae. The time elapsed since the beginning of the cycle is indicated in milliseconds. Scale bars: 5 mm. (C) Frames from a high-speed video showing the hair-like papillae (blue arrows) on the tongue of *P. discolor* in dorsolateral (left) and lateral (right) views. Scale bars: 1 mm. (D) Frame from a high-speed video showing the hair-like papillae (blue arrow) on the tongue of *A. geoffroyi*. Scale bar: 5 mm. (E) Scanning electron microscopy (SEM) image of the tongue of *P. discolor*, dorsal view. The blue arrow points to hair-like papillae, the yellow arrow points to horny papillae. Scale bars: 1 mm. (F) SEM image of the tongue of *A. geoffroyi*, dorsal view. The blue arrow points to hair-like papillae, the yellow arrow points to horny papillae. Scale bars: 1 mm.

*Phyllostomus discolor* and *A. geoffroyi* employed a lapping mechanism consisting of repetitive tongue protrusion and retraction cycles (e.g. Figs 1A,B; Fig. S2, Movies 1 and 2). Once *P. discolor* started feeding, it protruded its tongue and dipped it into the nectar, with the tongue stretched and flattened dorsoventrally, in a curved ventral motion. At maximum extension on each lick, the flattened tongue was folded inwards with the raised edges forming a partial channel medially and with the tongue tip further curving caudally. This curvature creates a kind of hook on the ventral side of the tongue tip (Fig. S2). Similar tongue bending was observed in *A. geoffroyi* individuals; however, their tongues were more mobile and bent in multiple directions and with less consistency (e.g. Fig. 1B,D), and did not flatten in the same manner.

An interesting difference in the dynamic tongue shapes between these species is the formation of the partial channel in *P. discolor* at the center of the tongue on the dorsal surface by muscular action that generates a local bending in the sagittal plane of the tongue (Fig. 1A). The tongue does not normally present a trough on the dorsal surface (Fig. 1E); this channel is formed as the tongue is pulled out during feeding. During most of the tongue protrusion, the medial trough is not visible; it progressively becomes longer and deeper near maximum protrusion, and persists during retraction (Fig. 1A). Additionally, in the lateral part of the tongue of *P. discolor*, we could see dilation of blood vessels as the tongue was extended (Fig. S2). In some cases, *P. discolor* individuals took nectar from the nectar surface (instead of fully submerging the tongue), bending and folding the tongue before barely touching the surface; this was more common in hand-held videos and sometimes at the beginning of some free-flying visits.

We observed hair-like papillae extending from the lateral surface of the tongue of *P. discolor* individuals during nectar extraction (Fig. 1C). Hair-like papillae were also observed in the high-speed videos of *A. geoffroyi* during feeding (Fig. 1D). Measurements from SEM images indicate that the hair-like papillae of *A. geoffroyi* are approximately twice as long as those of *P. discolor* (Fig. 1E,F). The tongues of both species show hydrophilic properties; when the tongue meets the nectar and retracts, a layer of liquid adheres to the tongue on the dorsal and lateral sides, which is then transported into the mouth (Fig. S3). During this process, hair-like papillae visibly separate from the tongue surface in both species, increasing their exposure to the nectar. The hair-like papillae of *P. discolor* are located along the sides of the tongue proximal to the horny papillae, which are located near the tongue tip (Fig. 1E). These hair-like papillae become longer and more abundant towards the middle section of the *P. discolor* tongue (Fig. 1E). These longer hair-like papillae will potentially be more exposed during formation of the ventro-caudal hook by the bending of the tip.

**Fig. 2.**
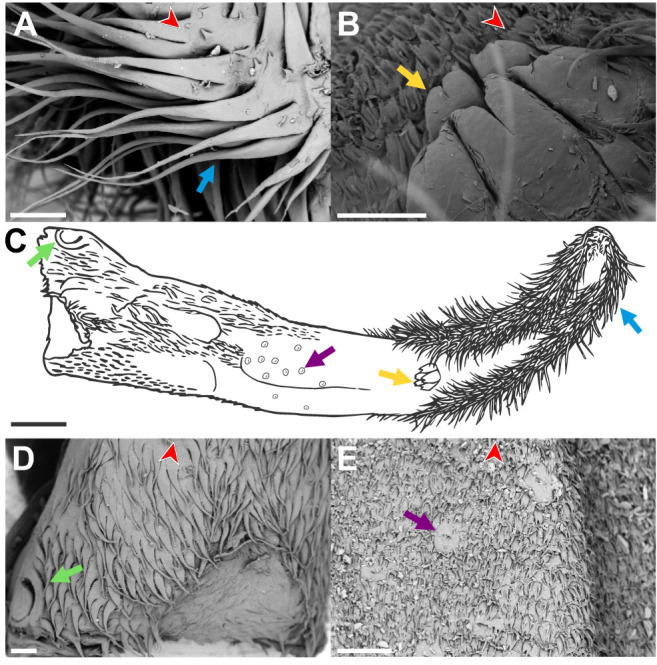
**SEM images of the dorsal tongue papillae of *A. geoffroyi*.** The red arrowheads point to the tip of the tongue. (A) Hair-like papillae (blue arrow). (B) Horny papillae (yellow arrow) surrounded by branched filiform papillae. (C) Schematic drawing of the dorsal surface of the tongue of *A. geoffroyi*, based on gross morphology and SEM observations; the tongue tip is oriented to the right. Green and blue arrows as in D and E. (D) Single-pointed filiform papillae and circumvallate papillae (green arrow). (E) Fungiform papillae (purple arrow) surrounded by branched filiform papillae. Scale bars: 200 µm in A, B, D and E; 1 mm in C.

In *A. geoffroyi*, hair-like papillae are also present along the tongue sides but, as opposed to *P. discolor*, most of them are located distal to the horny papillae, covering the lingual surface all the way to the tongue tip, where they are highly abundant (Fig. 1F). The hair-like papillae of *P. discolor* are shorter and less abundant than those of *A. geoffroyi* (see Quinche et al., 2023, for a deeper discussion of the lingual papillae of *P. discolor*). We observed six types of papillae in *A. geoffroyi*: four mechanical papillae – hair-like filiform papillae, horny papillae, single-pointed filiform papillae and branched filiform papillae; and two gustatory papillae – circumvallate papillae and fungiform papillae (Fig. 2).

**Fig. 3.**
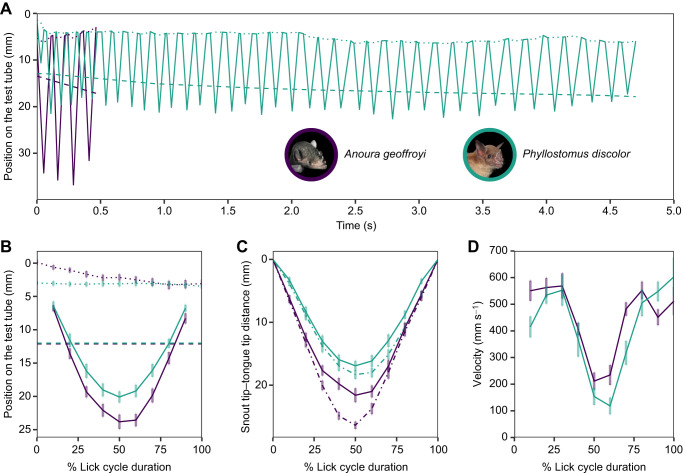
**Tongue movement pattern and kinematics for *P. discolor* (green) and *A. geoffroyi* (purple).** (A) Position on the nectar container of the snout tip (dotted line), tongue tip (solid line) and nectar depth (dashed line) for one complete visit through time. (B) Position on the nectar container of the snout tip (dotted line), tongue tip (solid line) and nectar depth (dashed line) for the first lick of multiple visits as a function of time (shown as percentage of the lick cycle). (C) Distance between the snout tip and the tongue tip in the *y*-axis (solid line) and following the curvature of the tongue (dot–dash line) for the first lick of multiple visits as a function of time (shown as percentage of the lick cycle). (D) Velocity of the tongue tip for the first lick of multiple visits as a function of time (shown as percentage of the lick cycle). In B–D, we show the mean±s.e.m. of the first lick of nine visits of four *P. discolor* individuals and 17 visits of five *A. geoffroyi* individuals.

**Fig. 4.**
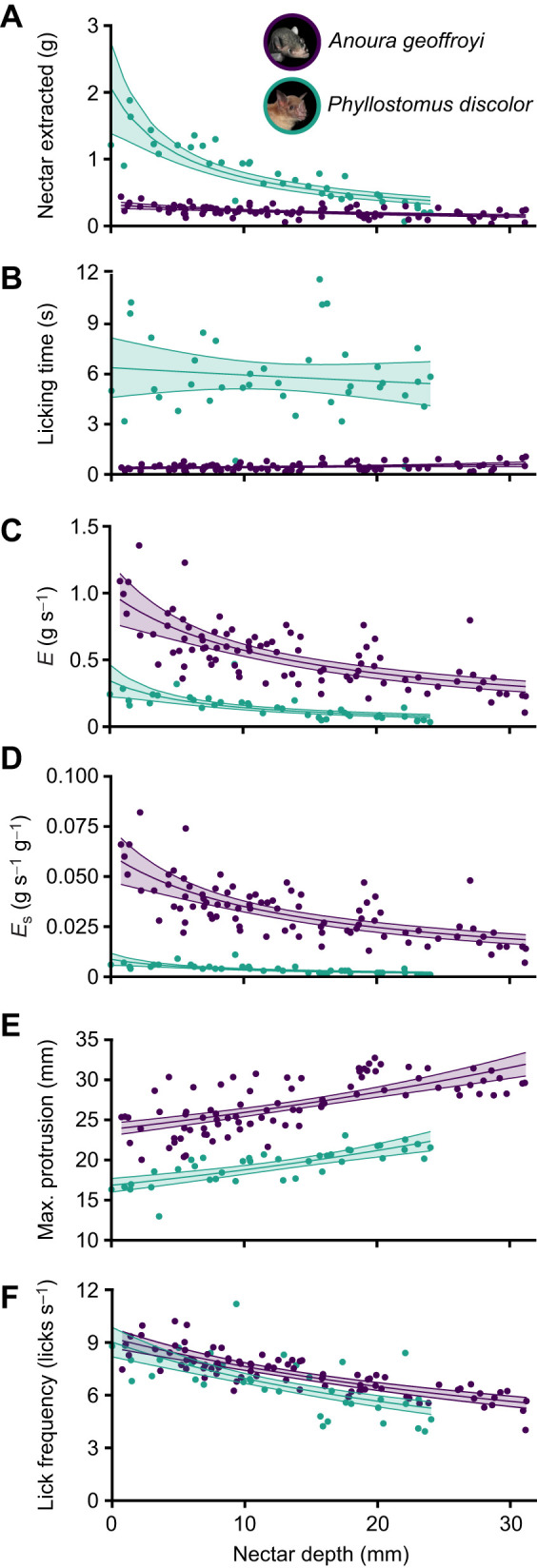
**Drinking behavior of *P. discolor* and *A. geoffroyi*.** Nectar depth at the beginning of the visit (defined as the vertical distance from the container rim to the nectar surface, with greater values indicating deeper nectar) versus (A) nectar extracted per visit, (B) licking time, (C) feeding efficiency (*E*), (D) size-adjusted (standardized) efficiency (*E*_s_), (E) maximum tongue protrusion and (F) lick frequency for complete visits. Individual points represent visit-level values calculated over the entire feeding visit: 40 visits of 10 *P. discolor* individuals and 88 visits of 11 *A. geoffroyi* individuals. The trend line represents the generalized linear model (GLM) between variables. The shaded area represents the 95% confidence interval of the model.

Although the two species exhibit similar (potentially convergent) hair-like papillae and tongue kinematics, we found differences between their two lapping mechanisms, as exemplified in the plot of tongue reciprocation patterns (Fig. 3). *Phyllostomus discolor* used more licks per visit, and the number of licks was more variable (4–77 licks) than in *A. geoffroyi* (1–7 licks) (e.g. Fig. 3A). Also, *P. discolor* continued feeding in most visits until it was unable to reach the nectar, making longer visits than *A. geoffroyi* (Figs 3A and 4B). Furthermore, snout insertion into the nectar container remained constant throughout the first lick cycle in *P. discolor*, in contrast to *A. geoffroyi*, which inserted the snout progressively toward the end of the first lick cycle (Fig. 3B). Additionally, we observed no unsuccessful attempts by *A. geoffroyi* to access nectar, unlike *P. discolor*, which occasionally missed the first one or two licks at the beginning of a visit. When this occurred, individuals moved their snout around while repeatedly extending their tongue to locate the nectar. Once they made contact with the nectar, they stopped adjusting their snout position and continued feeding. As expected, tongue tip insertion in *A. geoffroyi* was greater than that in *P. discolor* (mean±s.e.m. *A. geoffroyi*: 23.815±1.078 mm; *P. discolor*: 20.094±0.843 mm; nectar depth range 8–16 mm; Fig. 3B). The duration of protrusion and retraction in the two species was similar, with both species reaching maximum tongue protrusion at around 50% of the lick total duration (Fig. 3B).

Fig. 3C represents the curvature of the tongue as the difference between the total protrusion of the tongue (dot–dash line) and the straight distance between the tongue tip and snout tip (solid line). Both species curved their tongues during feeding and reached maximum curvature around maximum tongue protrusion (around 50% of the cycle). In general, *A. geoffroyi* curved its tongue more than *P. discolor*. In addition, the pattern of curvature throughout the cycle was different for the two species: *P. discolor* curved the tongue from 30% of the lick cycle to the end of the lick, retracting the curved tongue towards the mouth, whereas *A. geoffroyi* curved it from approximately 20% to 60% of the lick, retracting the tongue straight towards the mouth (Fig. 3C). Finally, Fig. 3D shows that the pattern of change in velocity during protrusion was similar for the two species; however, *A. geoffroyi* showed a higher retraction velocity than *P. discolor* (Fig. 3D). The change in tongue velocity during protrusion and retraction (slope) was similar for the two species, indicating a symmetrical pattern of tongue movement during feeding (Fig. 3D).

### Drinking behavior

To compare different metrics of nectar feeding behavior, we extracted data from 40 visits of 10 *P. discolor* individuals and 88 visits of 11 *A. geoffroyi* individuals. Both explanatory variables, species and nectar depth (defined as the vertical distance from the container rim to the nectar surface), had a significant effect on the amount of nectar extracted, but not their interaction (Table 1). The amount of nectar extracted per visit decreased with increasing nectar depth (i.e. nectar positioned farther below the rim) in both *A. geoffroyi* and *P. discolor* (Fig. 4A; Table S1). While *P. discolor* initially extracted more nectar per visit, its extraction rate declined more steeply with increasing depth compared with *A. geoffroyi*. However, the interaction term in Table 1 shows that this difference in slopes was not statistically significant. Nectar extraction per lick differed significantly between species (Wilcoxon rank-sum test, *W*=3499, *P*<0.001), with *A. geoffroyi* extracting more nectar per lick than *P. discolor*. Across all nectar depths, *A. geoffroyi* extracted on average 72.8±2.6 mg per lick (range: 26–146 mg), whereas *P. discolor* extracted 20.4±1.2 mg per lick (range: 7–42 mg). We found significant effects of species and nectar depth on the licking time, as well as their interaction (Table 1). At all nectar depths, *A. geoffroyi* visited the feeder for a much shorter time than *P. discolor* (Fig. 4B; Table S1). Licking time in *A. geoffroyi* showed a small depth-dependent change, whereas licking time in *P. discolor* remained relatively constant across nectar depths, resulting in a significant species×depth interaction (Table 1). Species, nectar depth and their interaction also significantly affected size-adjusted (standardized by body mass) efficiency (Table 1). The efficiency and size-adjusted efficiency decreased significantly with increasing nectar depth for both species (Fig. 4C,D; Table S1). However, this decrease was more pronounced for *P. discolor* than for *A. geoffroyi*. Nectar depth, species and their interaction significantly affected maximum tongue protrusion (Fig. 4E, Table 1). The mean maximum tongue protrusion was higher in *A. geoffroyi* than in *P. discolor* at all nectar depths (Fig. 4E; Table S1). Although for both species the maximum protrusion seemed to plateau when reaching deep nectar (suggesting an anatomical limitation), it is important to note that we did not experimentally push *A. geoffroyi* to its maximum tongue extension during feeding (i.e. we did not continue testing its ability to reach deeper nectar until it could no longer do so). Therefore, *A. geoffroyi*'s maximum tongue length could potentially exceed the length reported here. Nectar depth had a significant effect on lick frequency; however, species did not (Fig. 4F, Table 1). The lick frequency of the complete visits was similar for the two species, although lower (but without reaching significance) at almost all depths for *P. discolor* (Table 1).

**Table 1.** Results of generalized linear models (GLMs) evaluating the effects of species, nectar depth and their interaction on various drinking behavior metrics

Independent variable		Estimate	s.e.	*t*-value	*P*-value
Nectar extracted	Intercept	3.1709	0.2728	11.625	<2E−16*
	Species	−2.6866	0.2847	−9.435	2.94E−16*
	Nectar depth	0.1133	0.0208	5.455	2.55E−07*
	Species×Nectar depth	−0.0241	0.0230	−1.05	0.296
Licking time	Intercept	2.6645	0.2054	12.973	<2E−16*
	Species	−2.5079	0.2066	−12.139	<2E−16*
	Nectar depth	−0.0322	0.0111	−2.897	0.00446*
	Species×Nectar depth	0.0334	0.0112	2.969	0.00359*
Efficiency	Intercept	0.9940	0.1159	8.577	3.32E−14*
	Species	1.9183	0.5353	3.583	0.000486*
	Nectar depth	0.0751	0.0101	7.455	1.35E−11*
	Species×Nectar depth	0.3341	0.0585	5.715	7.72E−08*
Standardized efficiency	Intercept	16.4256	1.8712	8.778	1.11E−14*
	Species	98.0832	20.1982	4.856	3.53E−06*
	Nectar depth	1.2078	0.1618	7.464	1.29E−11*
	Species×Nectar depth	14.4617	2.2046	6.56	1.31E−09*
Max. protrusion	Intercept	0.0425	0.0007	63.87	<2E−16*
	Species	0.0137	0.0008	17.21	<2E−16*
	Nectar depth	−0.0004	0.0000	−10.12	<2E−16*
Lick frequency	Intercept	0.1081	0.0034	32.164	<2E−16*
	Species	0.0028	0.0062	0.441	0.6599
	Nectar depth	0.0023	0.0002	9.619	<2E−16*
	Species×Nectar depth	0.0010	0.0005	2.059	0.0416*

Response variables were nectar extracted, licking time, feeding efficiency, size-adjusted (standardized) efficiency, maximum tongue protrusion and lick frequency. For each model, the table shows the estimated effect (Estimate), standard error (s.e.), *t*-value and *P*-value for each predictor. Significant effects (*P*<0.05) are marked with an asterisk.

## DISCUSSION

### Feeding mechanism and associated morphology

We investigated the nectar extraction mechanism in two bat species that differ in their degree of phenotypic specialization for nectar feeding: *Anoura geoffroyi*, a glossophagine bat characterized by an elongated, highly protrusible tongue with specialized papillae and a hover-feeding strategy, and *Phyllostomus discolor*, which lacks many of the morphological and behavioral traits associated with specialized nectar feeding and typically feeds while perching. Both species employ a lapping technique, characterized by stereotypic reciprocating movements of their long and highly mobile tongues (Fig. 1A,B; Movies 1 and 2). The tongues of *P. discolor* and *A. geoffroyi* possess hair-like papillae that increase the surface area for liquid coating via adhesion, and thus function in this brush-tongue lapping technique, a widespread mechanism among nectar-feeding animals (Cuban et al., 2022; Harper et al., 2013; Hewes et al., 2023; Richardson and Wooller, 1986; Yang et al., 2014). However, differences in the relative positions and structures of these papillae (Fig. 1E,F) suggest distinct anatomical origins and analogous adaptations for nectar collection. Despite these differences, the shared lapping mechanism highlights convergence in their general nectar-feeding strategies.

In Glossophaginae bats, nectar is trapped between the hair-like papillae on the tongue during each lap (Harper et al., 2013). For *P. discolor*, the tongue's backward bending and central folding create a channel that increases nectar capture by optimizing the brush-like papillae's contact with nectar. This curving mechanism, observed in both species, likely enhances their ability to ‘mop’ nectar inside flowers, maximizing extraction efficiency. Although the two species share the lapping mechanism, *P. discolor* exhibited less tongue insertion and curvature but more snout insertion and licks per visit compared with *A. geoffroyi* (Fig. 3B,C). These differences reflect *P. discolor*'s lesser morphological and behavioral specialization for nectar feeding. Its shorter and thicker tongue (Figs 1E,F and 3B) limits its ability to reach greater nectar depths or achieve more pronounced tongue curvature. In contrast, *A. geoffroyi* exhibits tongue traits characteristic of glossophagine nectar specialists, including a longer and more protrusible tongue with abundant distal hair-like papillae that enhance nectar contact and extraction during lapping (Gamba et al., 2025; Harper et al., 2013; Muchhala et al., 2024; Nicolay and Winter, 2006).

Foraging behavior may influence some of the feeding mechanism characteristics. We hypothesize that landing in *P. discolor* and hovering in glossophagines (e.g. *A. geoffroyi*) relate to the differences in snout insertion and the number of licks per visit (Fig. 3A,B). Hovering imposes energetic constraints on glossophagines because of its high energy demand (Winter, 1998), limiting visit duration. In contrast, *P. discolor* faces no such constraint, enabling longer visits with more licks. Additionally, *P. discolor* initiated visits after inserting its snout, whereas *A. geoffroyi* started licking before reaching the limit of insertion and progressively pushed its snout further, reducing licking time and enhancing its ability to extract nectar efficiently. Protruding the tongue before inserting the snout may improve efficiency but it could be a risky strategy because it could potentially increase the chances of missing the target. However, unlike for *P. discolor*, we did not observe any unsuccessful attempts by *A. geoffroyi* to access nectar; hence, we suggest that *A. geoffroyi* is better at locating nectar than *P. discolor*. Some studies have highlighted the ability of specialized nectar bats to effectively detect and locate nectar sources using sensory adaptations such as elongated facial vibrissae for close-range tactile guidance and echolocation cues for flower classification and localization (Amichai et al., 2023; Gonzalez-Terrazas et al., 2016).

We observed similarities between the two species in tongue protrusion and retraction duration, as well as tongue velocity during the lick cycle (Fig. 3D). Tongue reciprocation followed a symmetrical path, with the two species exhibiting comparable velocity changes during protrusion and retraction. However, *A. geoffroyi* moved its tongue faster (with the starkest difference during retraction) than *P. discolor*, likely as a result of specialized vascular and muscular adaptations to feed on nectar in Glossophaginae species (Griffiths, 1978; Harper et al., 2013). Interestingly, this did not translate into higher licking rates; instead, it allowed *A. geoffroyi* to reach deeper in the container while still maintaining similar licking rates to *P. discolor*. It is also noteworthy that the two species did not seem to modulate their tongue protrusion distance following the receding nectar meniscus (e.g. Fig. 3A); instead, they started immersing their tongues completely in the nectar from the start of the visit.

### Drinking behavior

All evaluated feeding behavior variables differed significantly between species, except for licking rate. Nectar depth influenced all drinking behavior variables (Table 1). Both species extracted less nectar as depth increased (i.e. as nectar was positioned farther below the container rim), consistent with previous findings on Glossophaginae nectar-feeding bats (Gonzalez-Terrazas et al., 2012; Nicolay and Winter, 2006). This limitation arises from the lapping mechanism, where nectar adheres to the tongue's surface and is trapped between papillae (Harper et al., 2013). The extent of tongue submersion directly affects nectar extraction (Crompton and Musinsky, 2011; Reis et al., 2010). Additionally, the observed decrease in licking rate with increasing nectar depth may further constrain extraction efficiency, as fewer licks per unit time reduce the total amount of nectar obtained during a visit.

Interestingly, *P. discolor* extracted more nectar per visit than *A. geoffroyi* (Fig. 4A), despite the latter's specialized tongue morphology; however, this pattern reflects differences in body size and foraging strategy rather than greater feeding efficiency (see efficiency discussion below). *Phyllostomus discolor*’s foraging style (i.e. landing) allows it to forage on flowers for a longer period of time, which often involves draining flowers (Fischer, 1992; Heithaus et al., 1974; Pedrozo et al., 2018). This strategy may be advantageous but comes with increased predation risks and requires robust plant structures to support its weight; however, the larger body size of *P. discolor* may mitigate predation risk during prolonged, perched feeding compared with smaller bats. In contrast, when nectar extraction is evaluated on a per-lick basis, *A. geoffroyi* extracted significantly more nectar per lick than *P. discolor*, indicating greater efficiency of nectar uptake during individual licking cycles. Consistent with the higher nectar volume extracted per lick by *A. geoffroyi*, differences in filiform papillae length between species may influence nectar retention during individual licking cycles. The shorter filiform papillae of *P. discolor* (Quinche et al., 2023) may retain less nectar per lick than the longer distal filiform papillae of *A. geoffroyi* (Figs 1E,F and 2A; Nasto et al., 2018), particularly when extracting the last bits of nectar deep inside flowers.

The differences in foraging behavior between the two bat species significantly influenced their licking times (Figs 3A and 4B). In our experiments, *P. discolor* individuals remained perched and often continued feeding until it became challenging to access the remaining nectar. In contrast, the shorter licking times of *A. geoffroyi* are likely tied to the energetic demands of hovering during feeding (Winter, 1998). While the presence of a landing structure in our experimental setup may have extended the licking duration of *P. discolor*, making it longer than previously reported (Table S1) (Nicolay and Winter, 2006), we posit that our findings align with the species' natural foraging behavior, as several studies document that *P. discolor* commonly land or perch while feeding from flowers that provide such structures (Gribel et al., 1999; Hopkins, 1984; Pedrozo et al., 2018; Sazima and Sazima, 1977).

Nectar extraction efficiency, both standardized and non-standardized by body mass, was significantly lower in *P. discolor* compared with *A. geoffroyi* (Fig. 4C,D), consistent with previous findings comparing glossophagine and non-glossophagine species (Nicolay and Winter, 2006). This difference in efficiency is influenced by the varying degrees of morphological and behavioral specialization for nectar feeding between the species, including tongue length and the size and density of hair-like papillae (Fig. 1E,F), as well as the faster tongue motion (covering more distance in the same amount of time; Fig. 3A,D). Similar to earlier studies with glossophagines and lonchophyllines (Gonzalez-Terrazas et al., 2012; Tschapka et al., 2015), our results show that nectar depth has a significant negative effect on the extraction efficiency of *P. discolor* (Table 1). Additionally, overall efficiency in our experiments was lower than previously reported (Table S1) (Nicolay and Winter, 2006), likely because the landing structure allowed longer visits, reducing the amount of nectar extracted per unit time. While this strategy may lower efficiency by extending licking duration, it likely reduces the energetic cost of feeding compared with hovering (Voigt, 2004). Nonetheless, energetic costs associated with landing and take-off also need to be accounted for when comparing the overall energetics of these feeding strategies.

**Fig. 5.**
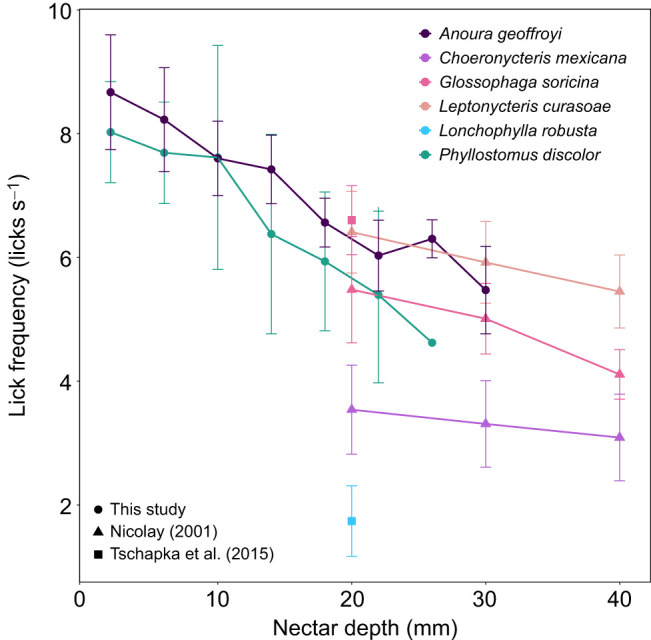
**Licking frequency decreases with nectar depth across nectar-feeding bat species.** Nectar depth (defined as the vertical distance from the container rim to the nectar surface, with greater values indicating deeper nectar) is plotted against licking frequency. Colors denote species; symbols denote data source [this study, Nicolay (2001) and Tschapka et al. (2015)]. Data points represent means±s.d. Sample sizes (number of individuals) were: *Choeronycteris mexicana n*=8, *Leptonycteris curasoae n*=7, *Glossophaga soricina n*=10 (from Nicolay, 2001) and *n*=4 (from Tschapka et al., 2015), *P. discolor n*=10, *A. geoffroyi n*=11 and *Lonchophylla robusta n*=3.

Our licking frequency results align with broader mammalian patterns showing an inverse relationship between body size and lapping rate, independent of the specific lapping mechanism employed (Gart et al., 2015; Olson, 2020; Reis et al., 2010; Weijnen, 1998). The notable exception is *Lonchophylla robusta*, whose non-lapping pumping mechanism produces much lower rates (1.74±0.57 licks s^−1^, *n*=3; Tschapka et al., 2015). Both species in our study achieved the highest lapping frequencies reported for bats to date, reaching 8–9 licks s^−1^ with only marginally higher rates in the specialized *A. geoffroyi* (Fig. 5). These peak frequencies occurred at shallow nectar depths not previously explored in other studies, which explains the higher maximum rates observed, given that licking frequency decreases as nectar depth increases because of the greater distance the tongue must travel to reach nectar positioned farther below the opening.

Across nectar-feeding bat species, licking frequency shows a consistent decline with nectar depth, with remarkably similar slopes between our study species and previously studied congeners (Fig. 5). However, while *P. discolor* and *A. geoffroyi* maintain comparable licking frequencies, their tongue protrusion distances differ dramatically, with the specialized *A. geoffroyi* achieving significantly greater extension (Fig. 4E). Given that tongue immersion also decreases with increasing meniscus depth (Fig. S4), we surmise that the decrease in extraction efficiency is not only due to increased tongue travel distance, and therefore decreased licking rate, but also due to a reduced contact area between the tongue and the nectar, which is particularly important in the lapping mechanism that relies on liquid adhesion to the tongue surface and entrapment among the hair-like papillae (Fig. 1). Future research should investigate the muscular mechanisms controlling both protrusion distance and licking rate, as well as sensory feedback to compensate for reduced tongue immersion by increasing protrusion. The ability of *P. discolor* to match the licking frequencies of highly specialized nectar bats despite its comparatively less specialized morphology warrants further investigation into the muscular adaptations underlying this convergent performance.

Our *A. geoffroyi* feeding performance data align well with published results for two congeners, *Anoura cultrata* and *Anoura caudifer* (Gamba et al., 2025), showing comparable values across all measured variables (Table S1). *Anoura geoffroyi* most closely resembles *A. cultrata* in feeding parameters, particularly visit time, while *A. caudifer* exhibited longer visits. Critically, all three species demonstrated consistent negative effects of nectar depth on feeding performance, supporting the generality of this constraint across closely related species sharing similar morphological adaptations for nectar feeding, including lapping mechanisms and tongue length (Muchhala et al., 2024). The consistency of these patterns across different studies and experimental setups demonstrates that closely related species with comparable tongue morphologies face similar physical constraints when accessing nectar at increasing depths.

Although dietary specialization enables species to exploit specific resources more efficiently, it comes with a tradeoff: specialization can limit the ability to utilize alternative resources when the preferred food is scarce (Rex et al., 2010). For *P. discolor*, the ability to switch to alternative food resources has been documented in various diet studies (Bonaccorso, 1979; Kwiecinski, 2006). The morphological, biomechanical and behavioral differences between *P. discolor* and *A. geoffroyi* influence the types of resources they can access and feed on effectively (Freeman, 1995; Gonzalez-Terrazas et al., 2012; Winter and von Helversen, 2003). The foraging strategy and tongue morphology of each species largely determine and restrict the types of flowers and other food items they can exploit. For example, the landing strategy and larger body size of *P. discolor* limit its access to smaller or more delicate flowers that cannot support its weight (Fischer, 1992; Gonzalez-Terrazas et al., 2012). Moreover, *P. discolor* is better suited for flowers with large openings and shorter corollas, as it relies more on snout insertion and possesses a wider, shorter and less flexible tongue compared with *A. geoffroyi* (Figs 1E,F and 2B,C). These phenotypic differences allow the two species to exploit different floral resources and coexist, even when nectar is an important food source for both species (Finke and Snyder, 2008).

### Conclusions and future directions

Our study highlights convergence in the nectar extraction mechanisms and associated morphology of the omnivorous *P. discolor* and the specialized nectar feeder *A. geoffroyi*. The two species employ an equally swift brush-tongue lapping technique, with the capture of nectar along the lingual surface augmented by the exposure of hair-like papillae. Notably, *P. discolor* demonstrates tongue mobility comparable to that of *A. geoffroyi*, as evidenced by their similar lick frequencies. At the same time, we found key contrasts in their feeding strategies, reflected in differences in tongue kinematics and nectar-feeding efficiency. This divergence is driven by morphological and behavioral differences, such as variation in tongue and papillae morphology and distinct foraging behaviors. Furthermore, *P. discolor* exhibits a combination of morphological and behavioral adaptations that enable efficient nectar exploitation while retaining the flexibility to feed on alternative resources, such as hard insects and fruits. This underscores the versatility of *P. discolor* and its capacity to adapt to diverse dietary niches.

Although *P. discolor* and *A. geoffroyi* share a brush-tongue lapping mechanism, it remains unclear whether the exposure or separation of papillae in either species is driven by a hemodynamic mechanism similar to that described for *Glossophaga soricina*, a glossophagine species (Harper et al., 2013). Future research should investigate this possibility, as well as determine the energetic costs associated with its feeding behavior, and compare these with other *Phyllostomus* species and nectarivorous bats. Exploring the trade-offs within and between traits involved in food acquisition could further illuminate the evolutionary shifts in feeding modes and levels of specialization among nectar-feeding bats.

## Supplementary Material

10.1242/jexbio.251404_sup1 Supplementary information
