# Supplementary material for: Convergent mechanisms, divergent strategies: a comparison of nectar intake between a generalist and a specialist bat species
Source: J Exp Biol. 2026 Mar 11;229(5):jeb251404. doi: 10.1242/jeb.251404 (PMC13006519; doi:10.1242/jeb.251404)
Supplement: Supplementary information [file jexbio-229-251404-s1.pdf]

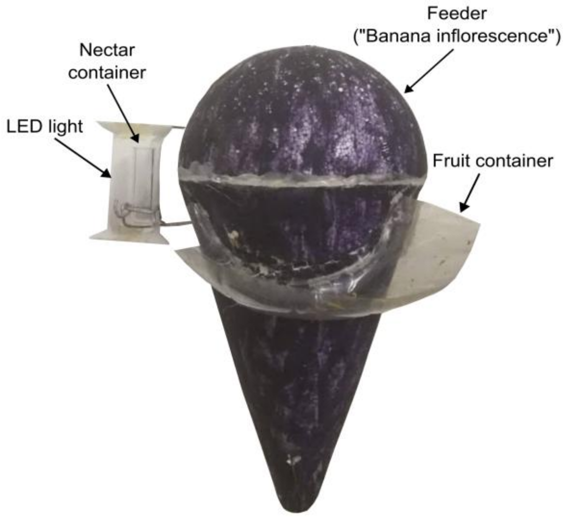

Fig. S1. Artificial feeder setup used in nectar-feeding experiments. Photograph of the banana-inflorescence–shaped feeder used during feeding trials, shown with the attached transparent, flat-sided nectar container (11 × 11 × 85 mm) and the LED light used for video illumination. The feeder was mounted on a tripod and attached to the base of the inflorescence.

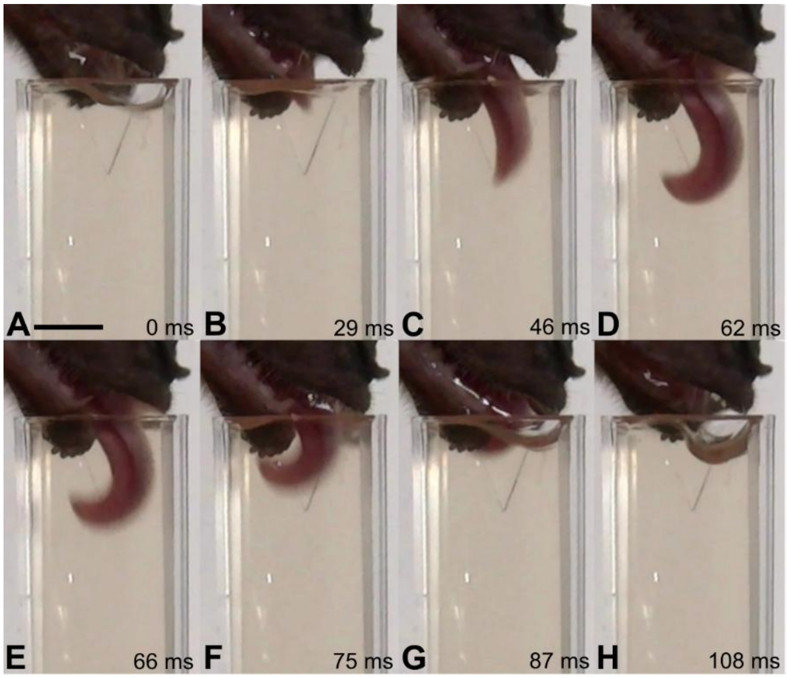

Fig. S2. Nectar extraction sequence of a single lick in *Phyllostomus discolor* (lateral view), illustrating the tongue's curvature into a 'hook' shape. Frames from a high-speed hand-held video. (A) start of the cycle. Bar: 5 mm. (B) and (C) protrusion. (D) maximum protrusion. Blood vessel dilatation is visible on the lateral side of the tongue. (E), (F), and (G) retraction. (H) end of the cycle. Time elapsed since the beginning of the cycle in milliseconds.

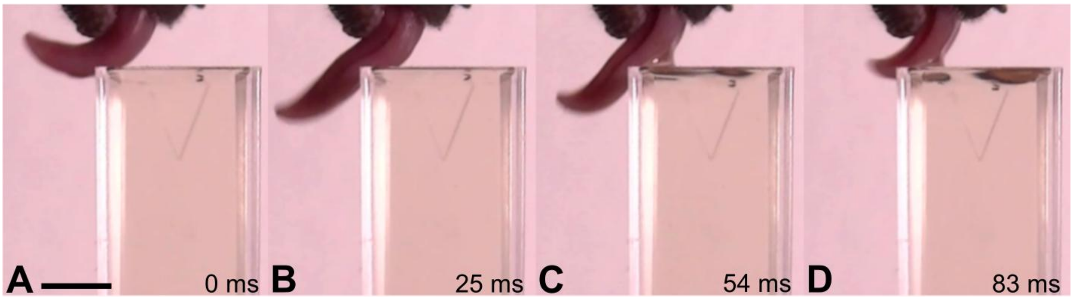

Fig. S3. Sequence of nectar extraction in *Phyllostomus discolor* (lateral view), illustrating the tongue's hydrophilic properties. Frames from a high-speed hand-held video. (A) protrusion. Bar: 5mm. (B) maximum protrusion. The lateral line of blood vessel dilatation is much more evident (C) and (D) retraction, nectar layer is forming on the dorsal part of the tongue. Time elapsed since the beginning of the cycle in milliseconds.

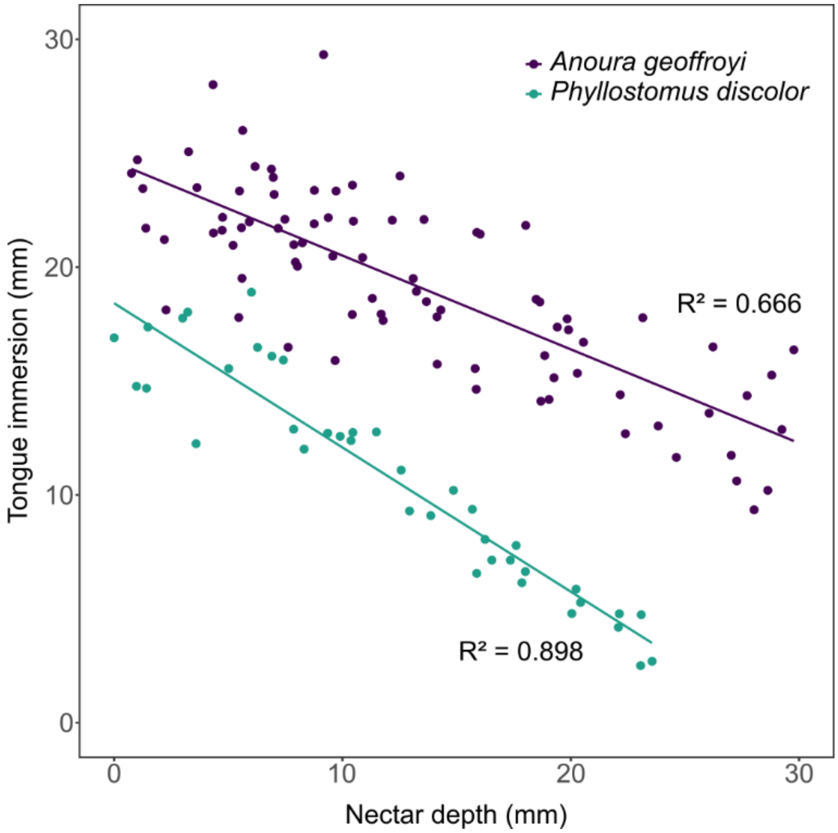

Fig. S4. Relationship between nectar depth and tongue immersion in *Anoura geoffroyi* and *Phyllostomus discolor*. Each point represents a single visit. Lines show fitted linear regressions (*P. discolor*: y = –0.651x + 18.558; *A. geoffroyi*: y = –0.447x + 24.964). 39 visits of 10 *P. discolor* individuals and 86 visits of 11 *A. geoffroyi* individuals.

Table S1. Drinking behavior data for *Phyllostomus discolor* and *Anoura geoffroyi* from this study, compared with published data for *A. caudifer* and *A. cultrata* (Gamba et al., 2025), and *Phyllostomus discolor* (Nicolay, 2001; Nicolay & Winter, 2006). n indicates the number of individuals per condition. In our study, nectar depth was treated as a continuous variable; values are averaged across specific depth intervals for comparison, while previous studies used fixed depths. Nectar extracted, licking/visit time, feeding efficiency, and size-adjusted efficiency are presented as mean ± SD. "Licking time" (this study) refers to the interval between the first and last visible tongue extension. In Nicolay (2001) and Nicolay & Winter (2006), "visit time" refers to the duration between snout entry and exit from the feeding tube. In Gamba et al. (2025), visit time is defined as the duration between snout insertion and withdrawal from the artificial flower. Feeding efficiency = nectar extracted / licking or visit time; size-adjusted efficiency = feeding efficiency / body mass. Feeding container internal width in this study: 11 mm; feeding tube diameters: 11 mm (Gamba et al., 2025) and 16 mm and 26 mm (Nicolay & Winter, 2006).

Species	Source	n	Meniscus before visit (mm)	Nectar extracted (g)	Licking/Visit time (s)	Feeding efficiency (g/s)	Size-adjusted feeding efficiency (g/s/g)
Anoura geoffroyi	This study	11	0 – 4	0.323 ± 0.074	0.391 ± 0.14	0.901 ± 0.285	0.055 ± 0.017
			4 - 8	0.249 ± 0.057	0.416 ± 0.155	0.655 ± 0.192	0.04 ± 0.012
			8 - 12	0.229 ± 0.055	0.428 ± 0.175	0.578 ± 0.132	0.035 ± 0.008
			12 - 16	0.203 ± 0.085	0.448 ± 0.236	0.5 ± 0.171	0.031 ± 0.01
			16 - 20	0.194 ± 0.070	0.459 ± 0.225	0.469 ± 0.149	0.029 ± 0.009
			20 - 24	0.175 ± 0.091	0.514 ± 0.262	0.347 ± 0.094	0.021 ± 0.006
			24- 28	0.168 ± 0.074	0.472 ± 0.287	0.419 ± 0.191	0.026 ± 0.011
			28 - 32	0.158 ± 0.082	0.655 ± 0.321	0.242 ± 0.082	0.015 ± 0.005
Anoura caudifer	Gamba et al., 2025	5	15	0.162 ± 0.062	0.819 ± 0.438	0.216 ± 0.057	0.021 ± 0.006
		5	30	0.128 ± 0.058	1.059 ± 0.700	0.136 ± 0.052	0.013 ± 0.005
		5	45	0.036 ± 0.035	1.000 ± 0.881	0.033 ± 0.020	0.003 ± 0.002

Journal of Experimental Biology • Supplementary information

Anoura cultrata	Gamba et al., 2025	4	15	0.172 ± 0.073	0.470 ± 0.245	0.441 ± 0.346	0.026 ± 0.021
		4	30	0.154 ± 0.068	0.567 ± 0.317	0.326 ± 0.235	0.020 ± 0.015
		4	45	0.104 ± 0.057	0.753 ± 0.456	0.145 ± 0.046	0.009 ± 0.003
Phyllostomus discolor	This study	10	0 - 4	1.338 ± 0.336	6.569 ± 2.764	0.219 ± 0.044	0.006 ± 0.001
			4 - 8	1.196 ± 0.145	6.146 ± 1922	0.209 ± 0.062	0.005 ± 0.002
			8 - 12	0.798 ± 0..241	4.814 ± 2.019	0.211 ± 0.13	0.005 ± 0.003
			12 - 16	0.662 ± 0.114	7.058 ± 3.217	0.113 ± 0.055	0.003 ± 0.001
			16 - 20	0.508 ± 0.129	5.845 ± 2.514	0.094 ± 0.024	0.002 ± 0.001
			20 - 24	0.31 ± 0.132	4.934 ± 2.088	0.073 ± 0.032	0.002 ± 0.001
			24- 28	0.201	5.842	0.034	0.001
	Nicolay, 2001; Nicolay and Winter, 2006	5	20	1.103 ± 0.585	1.56 ± 0.54	0.710 ± 0.232	0.0168 ± 0.0054
		5	30	0.517 ± 0.128	1.37 ± 0.30	0.403 ± 0.152	0.0095 ± 0.0036
		5	40	0.322 ± 0.128	1.80 ± 0.88	0.225 ± 0.139	0.0054 ± 0.0034

Journal of Experimental Biology • Supplementary information

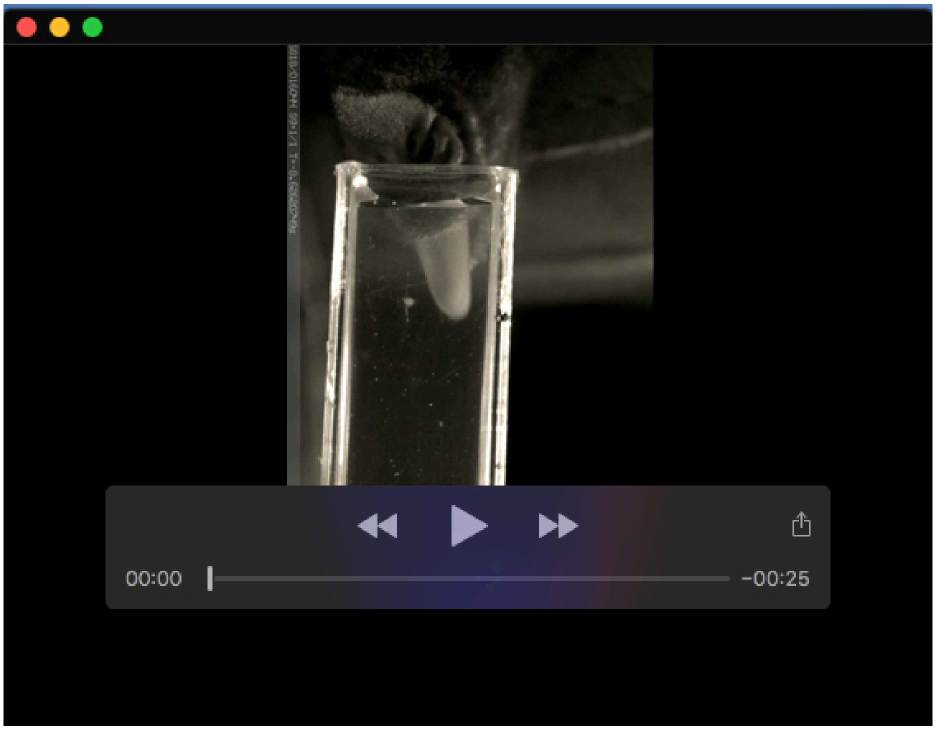

Movie 1. High-speed video of *Phyllostomus discolor* feeding on nectar. At maximum tongue extension during each lick, the tongue folds medially, and hair-like papillae extend away from the tongue surface. Recording rate=1000 fps; playback speed=20 fps.

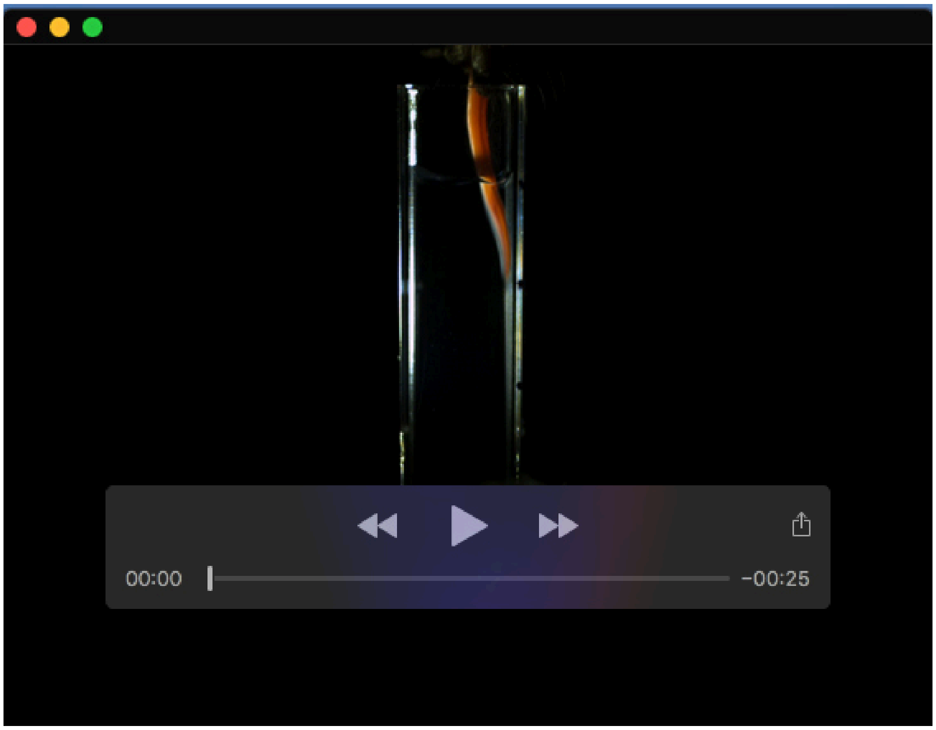

Movie 2. High-speed video of *Anoura geoffroyi* feeding on nectar. Note the extension of hair-like papillae and the tongue curving in multiple directions during nectar contact. Recording rate=500 fps; playback speed=12.5 fps.
